# Deep Brain Stimulation for Pantothenate Kinase-Associated Neurodegeneration

**DOI:** 10.1155/2015/245735

**Published:** 2015-02-23

**Authors:** Pedro J. Garcia-Ruiz, Joaquin Ayerbe, Lydia Vela Desojo, Cici E. Feliz, Javier del Val Fernandez

**Affiliations:** ^1^Department of Neurology, Fundacion Jimenez Diaz, Avenida Reyes Catolicos 2, 28040 Madrid, Spain; ^2^Department of Neurosurgery, Fundacion Jimenez Diaz, Avenida Reyes Catolicos 2, 28040 Madrid, Spain; ^3^Department of Neurology, Hospital Fundacion Alcorcon, Calle Valdelaguna 1, Alcorcón, 2892 Madrid, Spain

## Abstract

Pantothenate kinase-associated neurodegeneration (PKAN) is usually associated with dystonia, which is typically severe and progressive over time. Pallidal stimulation (GPi DBS) has been carried out in selected cases of PKAN with drug-resistant dystonia with variable results. We report a 30-month follow-up study of a 30-year-old woman with PKAN-related dystonia treated with GPi DBS. Postoperatively, the benefit quickly became evident, as the patient exhibited a marked improvement in her dystonia, including her writing difficulty. This result has been maintained up to the present. GPi DBS should be considered in dystonic PKAN patients provided fixed contractures and/or pyramidal symptoms are not present.

Neurodegeneration with brain iron accumulation (NBIA) includes a heterogeneous group of disorders characterized by iron accumulation in the brain [1]. The most common subtype of NBIA, pantothenate kinase-associated neurodegeneration (PKAN), is caused by mutations in the* PANK2* gene [1, 2]. Several phenotypes of PKAN can be recognized although movement disorders are generally present, especially dystonia, which is typically severe and progressive over time [2, 3]. Pallidal stimulation (GPi DBS) has been considered in selected cases of PKAN with drug-resistant dystonia, with results ranging from excellent to very modest improvement [4–10]. To date, most PKAN patients selected for GPi DBS, save some exceptions, already presented severe dystonia.

We report a 30-month follow-up study of a 30-year-old woman with PKAN-related dystonia treated with GPi DBS. At the age of 18 years she developed progressive writing difficulty due to focal dystonia, which remained her only complaint for several years. With the passage of time her dystonia also included her left arm, trunk, and cervical region and minimal overflow dystonia was also present in both legs. She did not develop spasticity, cognitive decline, impaired visual acuity, or any other problem. The patient was treated with several drugs including anticholinergics, tetrabenazine, and botulinum toxin, resulting in an improvement in her cervical dystonia while failing to ameliorate her difficulty with writing, her main functional problem. In March 2012 she scored 42/120 on the motor section and 13/30 on the disability section of the Burke-Fahn-Marsden dystonia rating scale (BFMDRS).

PKAN diagnosis was based on the findings of MRI images (typical tiger eye) and the detection of three mutations in the gene encoding pantothenate kinase 2 (PANK2): a previously described pathogenic mutation (cDNA C423T; AA: Thr 528 Met) and two unclassified variants (cDNA. C375G; AA Ala 492 Gly and cDNA G443C; AA Gly 555 Arg).

Due to her severe writing problems (which had made it impossible for her to write a sentence) and given that the previous medical treatment had been unsuccessful and based on previous cases, treatment with GPi DBS was proposed. After informed written consent was obtained, the patient underwent bilateral implantation of a quadripolar electrode into the GPi. The surgical procedure was performed by frame-based stereotactic technique while the patient remained under generalized anesthesia. The target was bilaterally defined by CT/MR image fusion with the standard coordinates for GPi (2 mm anterior to the midcommissural point and 20 mm lateral and 4 mm ventral to the intercommissural line). Neurophysiologic target verification was performed intraoperatively by simultaneous multielectrode microrecordings, microstimulation, and visual evoked potentials. After determining the definite bilateral coordinates, a permanent quadripolar electrode (DBS 3387, Medtronic) was implanted in each side. During the procedure, the electrodes were connected to a dual-channel pulse generator (Kinetra, Medtronic) subcutaneously placed in the right subclavicular area. The positioning of the electrodes in the caudal ventral portion of GPi was verified with MRI (Figures 1 and 2).

Postoperatively, the benefit quickly became evident, as the patient exhibited a marked improvement in her dystonia, including her writing difficulty; the patient regained the ability to write freely (see video in Supplementary Material available online at http://dx.doi.org/10.1155/2015/245735). Six months following the procedure (October 2012) the improvement was confirmed and the patient scored 26/120 and 8/30 on the BFMDRS scale. This improvement has been maintained up to the present, although occasionally she requires botulinum toxin injection in her left arm. Improved performance of activities of daily living was evident and she is presently able to cope with most daily living activities. The most recent BFMDRS scores were 24/120 and 9/30 on the BFMDRS scale (Table 1).

Currently, the electric parameters are as follows:

- right channel: pulse width 60 *μ*s; rate of 130 Hz, amplitude 3.2 V, case (+), and contact 0 (−),
- left channel: pulse width 60 *μ*s; rate of 130 Hz, amplitude 3.2 V, case (+), and contact 4 (−).

GPi DBS is a useful treatment for primary and secondary dystonia including PKAN [4–10]. The result is highly variable, since, in general terms, advanced PKAN cases are selected for GPi DBS [4–10], and there seems to be a relationship between baseline severity and postoperative result [7]. To date, our case represents a mild variant of PKAN, with late onset (>18 years) and very slow evolution since she has not developed any other manifestation except dystonia over time. Pure or relatively pure dystonia, even when associated with progressive neurodegenerative diseases, such as PKAN, may be good candidates for GPi DBS. It is worthwhile to take into account that PKAN can manifest as pure dystonia with a relatively benign clinical course [2, 8, 11]. In any case, as Adamovicová et al. [9] have already pointed out GPI DBS should be considered in dystonic PKAN patients provided fixed contractures and/or pyramidal symptoms are not present [9, 10].

## Figures and Tables

**Figure 1:**
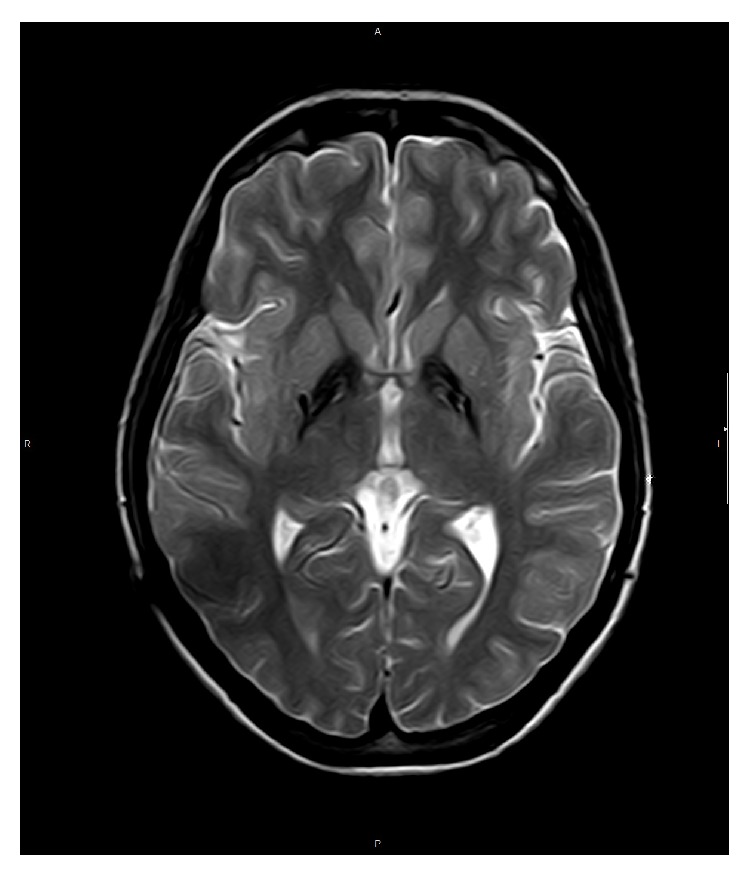


**Figure 2:**
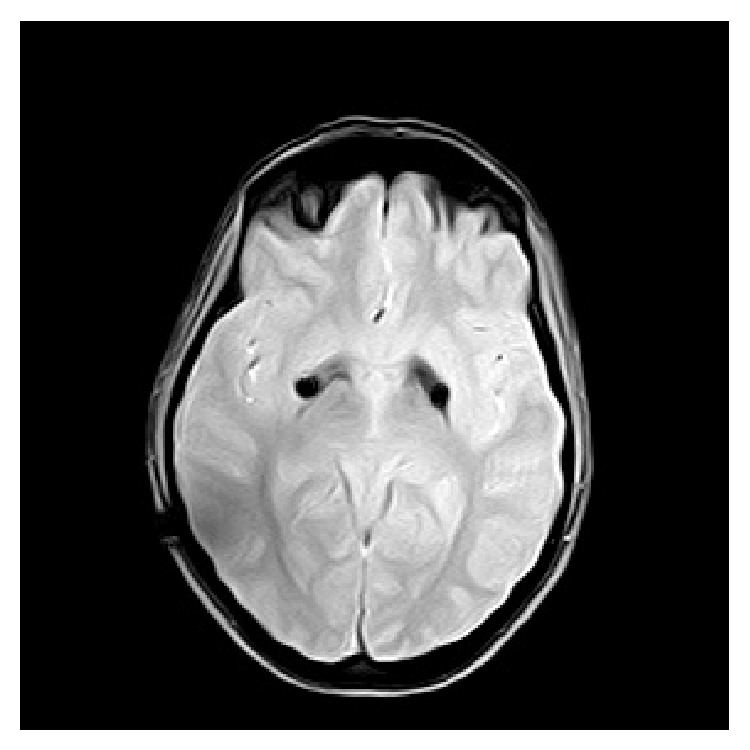


**Table 1:** Evolution of the BFMDRS (Burke-Fahn-Marsden dystonia rating scale) over time.

	Baseline preop	6 months	9 months	12 months	30 months
Motor section	42	26	22	22	24
Disability section	13	8	8	9	9
